# Pleomorphic carcinoma of the breast associated with cyst formation: a unique surgical case focusing on cytological and immunohistochemical findings. Cystic breast PC

**DOI:** 10.1186/1746-1596-8-75

**Published:** 2013-05-07

**Authors:** Sohsuke Yamada, Atsunori Nabeshima, Yoshika Nagata, Takashi Tasaki, Hirotsugu Noguchi, Shohei Kitada, Satoshi Kimura, Ke-Yong Wang, Shohei Shimajiri, Yasuyuki Sasaguri

**Affiliations:** 1 Departments of Pathology and Cell Biology, School of Medicine, University of Occupational and Environmental Health, 1-1 Iseigaoka, Yahatanishi-ku, Kitakyushu 807-8555, Japan; 2 Departments of Surgery II, School of Medicine, University of Occupational and Environmental Health, 1-1 Iseigaoka, Yahatanishi-ku, Kitakyushu 807-8555, Japan; 3 Departments of Bio-information Research Center, School of Medicine, University of Occupational and Environmental Health, 1-1 Iseigaoka, Yahatanishi-ku, Kitakyushu 807-8555, Japan

**Keywords:** Plemorphic carcinoma, Breast, Cytology, Cyst

## Abstract

A mammary nodular lesion was recognized one month before the surgery in the right upper breast of a 55-year-old female. The fine needle aspiration cytology specimens contained many individual bizarre, multi-nucleated, and/or giant cells having hyperchromatic pleomorphic nuclei, prominent nucleoli, and relatively abundant cytoplasm, admixed with numerous mitotic figures in a hemorrhagic or inflammatory background. A small amount of sheet-like or three-dimensional clusters of malignant cells coexisted. We first interpreted it as high-grade malignancy, such as invasive carcinoma, not otherwise specified. A right breast-conserving surgery was performed, and gross examination revealed a cystic cavity-formed and solid tumor lesion, measuring 35 × 35 × 25 mm and looking gray-yellowish to -whitish. On microscopic examination, the tumor was composed of a diffuse proliferation of highly atypical cells devoid of adhesive characteristics, including many multi-nucleated giant bizarre cells, in a haphazard fashion with stromal invasion, alternating with sarcomatoid features of spindle tumor cells. The cystic cavity was surrounded by hemorrhagic and inflammatory granulation tissue and lined by mostly denuded but atypical tumor cells or bland-looking flattened epithelial cells. Immunohistochemically, these tumor cells are specifically positive for all epithelial markers. Therefore, we made a conclusive diagnosis of pleomorphic carcinoma of the breast with cyst formation. We should be aware that, owing to its characteristic findings, cytopathologists can diagnose correctly, based on careful cytological examination of adequate samplings.

**Virtual slides:**

The virtual slide(s) for this article can be found here: http://www.diagnosticpathology.diagnomx.eu/vs/9290689448998782

## Background

An entity of mammary pleomorphic carcinoma (PC) was first established based on the following criteria, as previously described by Silver and Tavassoli in 2000 [1]: ≥50% of the tumor manifested a pleomorphic cell population, and tumors of lobular origin were excluded out. The World Health Organization (WHO) classification of tumours of the breast adopts this terminology and now records PC as a very rare variant of high-grade invasive carcinoma of no special type, characterized by proliferation of pleomorphic and bizarre giant cells comprising >50% of the tumor cells in a background of adenocarcinoma or adenocarcinoma with spindle and squamous differentiation [2]. PC is an extremely uncommon subtype of invasive breast carcinoma, and actually, one original paper described that less than 0.1% breast tumors were classified as PCs among a large series of surgically-resected cases [3]. Silver and Tavassoli have reported that the average PC tumor size was 5.4 cm, 52% and 12% of patients had axillary lymph node metastases and distant metastases, respectively, and 38% of them were dead of its disease (mean disease-specific postoperative survival: 22 months) [1]. Corresponding to most invasive ductal carcinomas of the breast, PC could be seen at any age, but tend to occur in the perimenopausal period [1,3], as in the present case. The authors have confirmed that PC is a unique entity with a significantly poor outcome [1], similar to PCs in the other organs, such as lung or small intestine [4,5]. However, PC of the breast potentially poses a diagnostic challenge to clinicians and cytopathologists, since its entity seems to be difficult to conclude a correct diagnose preoperatively. In fact, we recently have seen no series of large detailed studies regarding with cytological findings in PCs of the breast, except for few case reports previously published [6-8]. Hence, it is critical to establish an accurate preoperative diagnosis by fine needle aspiration cytology.

Here, we report a unique surgical case of PC of the breast, associated with cyst formation. Based on the cytology specimens, we preoperatively interpreted it merely as invasive carcinoma, not otherwise specified.

## Materials and methods

The patient was a 55-year-old Japanese woman. Fine needle aspiration cytology from the mammary nodule was performed, followed by a right breast-conserving surgery. The tumor specimens after fixation in 10% neutral buffered formalin were embedded in paraffin for histological or immunohistochemical examinations. All immunohistochemical stainings were carried out using Dako Envision kit (Dako Cytomation Co., Glostrup, Denmark) according to the manufacturer’s instructions, and using commercially available prediluted monoclonal antibodies against the following antigens: cytokeratins (Cam5.2; Becton Dickinson, San Jose, CA, USA, diluted 1:1, AE1/AE3; Chemicon International, Tamecula, CA, USA, diluted 1:200, CK5/6; Boehringer Ingelheim Pharma GmbH & Co. KG, Ingelheim, Germany, diluted 1:50, 34βE12; Leica Microsystems, Wetzlar, Germany, diluted 1:200), EMA (Dako, diluted 1:100), E-cadherin (Becton Dickinson, diluted 1:1,000), β-catenin (Becton Dickinson, diluted 1:100), estrogen receptor (ER; BioMed Immunotech, Inc., Tampa, FL, USA, diluted 1:25), progesterone receptor (PgR; Dako, diluted 1:6), HER2 protein (Dako, diluted 1:2), epidermal growth factor receptor (EGFR; Dako, diluted 1:1), p63 (Dako, diluted 1:30), S-100 protein (Dako, diluted 1:900), CD10 (NOVOCASTRA laboratories Ltd., Newcastle, United Kingdom, diluted 1:20), α-smooth muscle actin (α-SMA; Dako, diluted 1:150), calponin (Dako, diluted 1:50), vimentin (Dako, diluted 1:100), CD31 (Dako, diluted 1:20), CD34 (BioMed Immunotech, Inc., diluted 1:1), D2-40 (Dako, diluted 1:50), desmin (Dako, diluted 1:150), myogenin (Dako, diluted 1:30), c-kit (Santa Cruz Biotechnology, Inc., Santa Cruz, CA, USA, diluted 1:40), CD3 (Dako, diluted 1:1), CD4 (Dako, diluted 1:1), CD8 (Nichirei Biosciences Inc., Tokyo, Japan, diluted 1:1), CD20 (Dako, diluted 1:200), CD30 (Dako, diluted 1:40), CD45 (Dako, diluted 1:400), CD68 (KP-1; Dako, diluted 1:100), CD79a (Dako, diluted 1:50), bcl-2 (Dako, diluted 1:30), p53 (Dako, diluted 1:30), and Ki-67 (MIB-1; Dako, diluted 1:50). Since all tumor specimens were fixed in formalin, transmission electron microscopy could not be performed.

## Case presentation

The patient had no history of malignancy, immunosuppressive disorders, use of immunosuppressive medications, or unusual infections. Family history showed that her aunt also suffered from breast cancer and her father had colorectal cancer.

She first noticed a nodule in the right breast one month before the resection. On initial examination, a reddish swelling mass more than 3 cm in diameter was palpable mainly in the right upper medial breast. Laboratory data, including blood cell count, chemistry and tumor markers, were within normal limits, except for slightly increased carbohydrate antigen (CA) 15–3 level (35.1 U/mL). Ultrasound sonography showed an intracystic tumor lesion, measuring approximately 3.5 cm in diameter, consisted of a mixture of a heterogeneously enhanced solid component and a cystic component on chest CT scans. CT scans of the head, neck, chest, and abdomen disclosed no definite evidence of metastases in the lymph nodes or other organs. MRI revealed a heterogeneous hyperintense cystic mass on T_2_-weighted images, associated focally with a contrast-enhanced irregular wall portion. About 1 month after surgery, the patient has been medicated on adjuvant chemotherapy, consisting of four cycles of epirubicin (120 mg/body), cyclophosphamide (800 mg/body), and fluorouracil (800 mg/body), and will be followed up with irradiation. She had neither recurrence nor metastases of the breast cancer, respectively, and was alive and well at 10 months after the operation.

## Pathological findings

**Figure 1:**
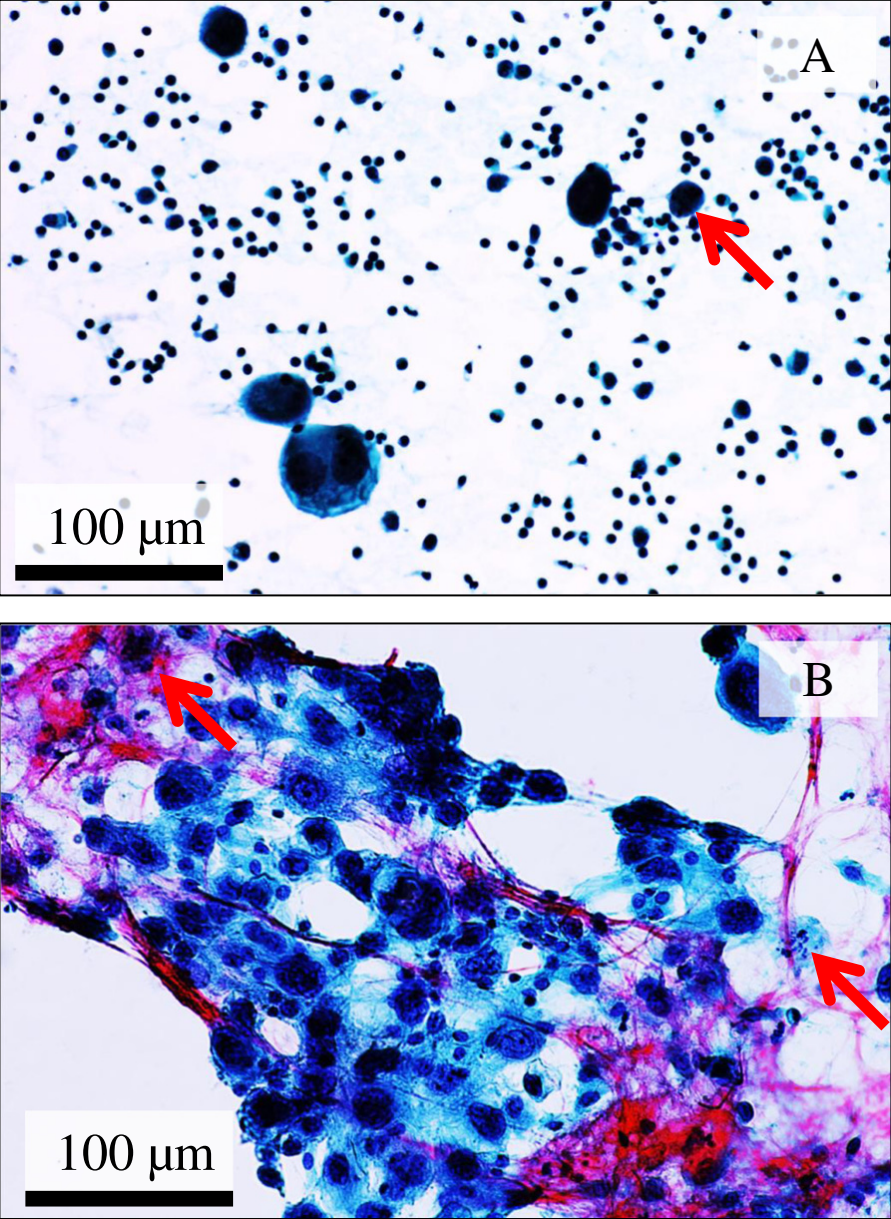
**Cytomorphologic examination of the fine needle aspiration cytology in PC specimens.** (**A**) The cytology specimens were consisted predominantly of many individual, scattered malignant giant bizarre cells having often multi-nucleated pleomorphic nuclei in a hemorrhagic or inflammatory background (Papanicolaou stains). Bar = 100 μm. (**B**) A small number of clusters of cohesive and sheet-like or three-dimensional malignant tumor cells coexisted. Those malignant cells showed very large (up to approximately 100 μm in diameter), pleomorphic, and bizarre or spindle nuclei and had relatively abundant cytoplasm. Additionally, the nuclei contained conspicuous nucleoli and numerous mitotic figures including atypical mitoses (arrows) (Papanicolaou stains). Bar = 100 μm.

The first fine needle aspiration cytology specimens were consisted mainly of many individual mono- or multi-nucleated bizarre giant cells (Figure 1A), coexisted with a small amount of clusters of cohesive and sheet-like or three-dimensional malignant cells (Figure 1B), in a hemorrhagic or inflammatory background (Figure 1A–B). The malignant tumor cells showed markedly large (up to approximately 100 μm in diameter), hyperchromatic pleomorphic, and round to oval or spindle nuclei and had relatively abundant cytoplasm (Figure 1A–B). Additionally, the nuclei often had prominent nucleoli and numerous mitotic figures, including atypical mitoses (Figure 1A–B). No malignant squamoid cells were seen. Based on that, we first interpreted it as high-grade malignancy, such as invasive carcinoma, not otherwise specified, and an ordinary right breast-conserving surgery was performed.

**Figure 2:**
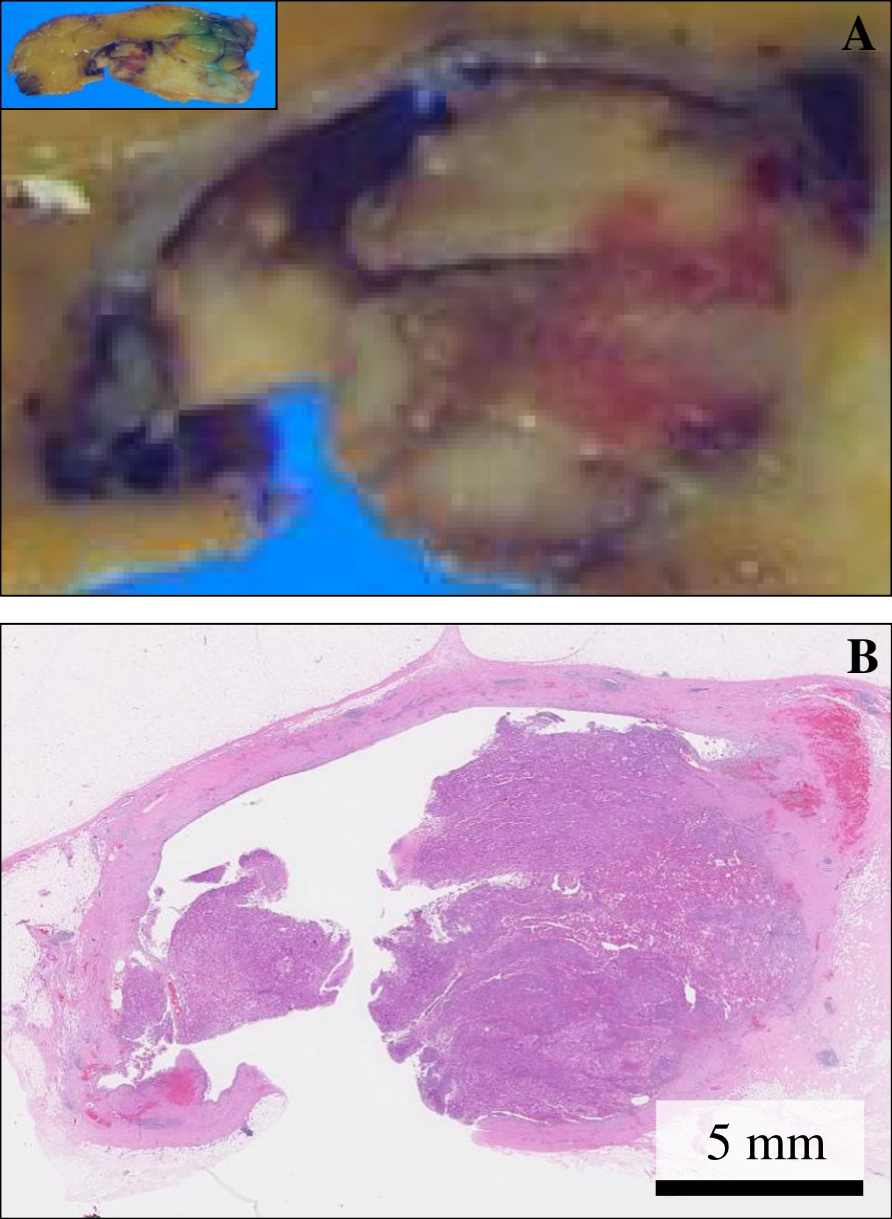
**Gross and microscopic examination of the resected PC specimen.** (**A**) On gross examination, the cut surface revealed a peripherally cystic cavity-formed, relatively well-defined, and solid firm mass, measuring 32 × 27 × 25 mm, which looked from gray-yellowish to -whitish in color. This cystic cavity measured approximately 15 × 10 mm, filled with hemorrhagic materials. Bar = 5 mm. (**B**) A scanning magnification (H&E stains) revealed that the cancer lesions were partly surrounded by the cystic cavity (lt. side) but irregularly involved its wall in part (rt. side). There were neither extensive components of intraductal carcinoma nor lobular carcinoma areas within our thorough investigation. Bar = 5 mm.

On gross examination, the cut surface revealed a peripherally cystic cavity-formed, relatively well-demarcated, and solid firm mass, measuring 32 × 27 × 25 mm, which looked from gray-yellowish to -whitish in color (Figure 2A). This cystic cavity measured approximately 15 × 10 mm, filled with hemorrhagic but not necrobiotic materials. The background of the breast had no remarkable change, e.g., not mastopathic. A scanning magnification of it showed that the cancer lesions, more than 60% in volume, were partly surrounded by the cystic cavity but irregularly involved its wall in part (Figure 2B). There were neither extensive components of intraductal carcinoma nor lobular carcinoma areas within our thorough investigation.

**Figure 3:**
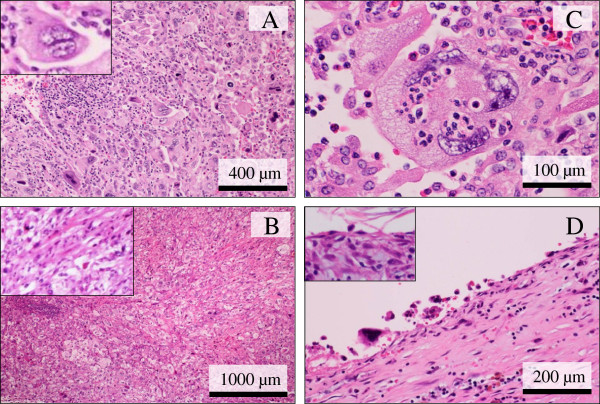
**Microscopic examination of the PC of the breast.** (**A**) Low power view showed a diffuse proliferation of large and highly atypical cells devoid of adhesive characteristics, including many multi-nucleated bizarre giant cells (inset), arranged predominantly in a haphazard fashion with a chronic and acute inflammatory infiltrate (H&E stains). Bar = 400 μm. (**B**) While, a small amount of sarcomatoid components, consisted mainly of atypical spindle cells (inset), coexisted. Our careful histological examination revealed neither evidences of squamous nor chondro-osseous metaplasia (H&E stains). Bar = 1,000 μm. (**C**) On high-power view, emperipolesis of inflammatory cells was sometimes recognized (H&E stains). Bar = 100 μm. (**D**) The cystic cavity was surrounded by hemorrhagic and inflammatory granulation tissue and lined by mostly denuded but single atypical giant bizarre tumor cells or bland-looking flattened epithelial cells (inset) (H&E stains). Bar = 200 μm.

Microscopic findings demonstrated a diffuse proliferation of large and highly atypical cells devoid of adhesive characteristics, having hyperchromatic pleomorphic nuclei and relatively abundant eosinophilic or clear cytoplasm, admixed with many multi-nucleated bizarre giant cells and numerous mitotic figures including atypical mitoses, arranged predominantly in a haphazard fashion with a chronic and acute inflammatory infiltrate (Figure 3A). By contrast, a small amount of sarcomatoid components, consisted mainly of atypical spindle cells, were coexistent with it (Figure 3B). Apparent keratinization, intercellular bridge, or intracytoplasmic mucin was not evident. Our careful histological examination revealed neither evidences of squamous nor chondro-osseous metaplasia. On high-power view, emperipolesis of inflammatory cells was uniquely, sometimes seen (Figure 3C). Moreover, the cystic cavity was surrounded by hemorrhagic and inflammatory granulation tissue and lined by mostly denuded but atypical giant tumor cells or bland-looking flattened epithelial cells (Figure 3D). The tumor cells partly involved the adjacent cyst wall but without evidence of apparent vessel permeation. Neither involvements of fat nor skin were recognized.

**Figure 4:**
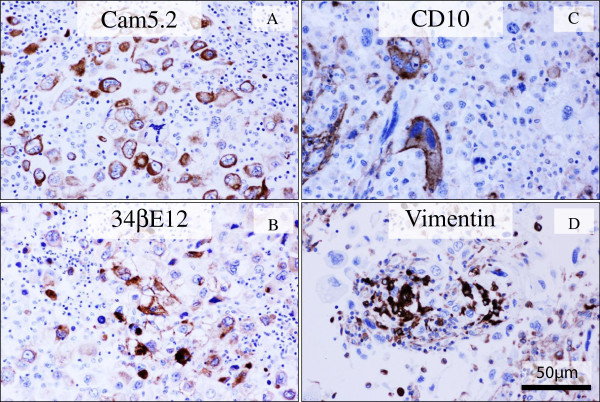
**Immunohistochemical examination of the PC of the breast.** (**A, B, C, D**) The giant bizarre tumor cells of PC were specifically positive for Cam5.2 (**A**) and 34βE12 (**B**), and focally positive for CD10 (**C**). Additionally, one part of sarcomatoid spindle tumor cells were positive for vimentin (**D**). Bars = 50 μm.

Immunohistochemically, these highly atypical giant cells were specifically positive for all epithelial markers, i.e., Cam5.2 (Figure 4A), AE1/AE3, CK5/6, 34βE12 (Figure 4B), and EMA, and focally positive for CD10 (Figure 4C), CD4, and EGFR, whereas negative for E-cadherin, β-catenin, p63, S-100 protein, α-SMA, calponin, CD31, CD34, D2-40, desmin, myogenin, c-kit, CD3, CD8, CD20, CD30, CD45, CD68, CD79a, bcl-2, and p53. Additionally, sarcomatoid spindle cells were focally positive for vimentin (Figure 4D). On the other hand, these tumor cells were completely negative for hormone receptors (ER and PgR) and HER2, manifesting as triple-negative breast cancer. MIB-1 labeling index was approximately 30% in the proliferating malignant cells. The above tumor-surrounding inflammatory cells were CD3-positive and CD4-predominant T-lymphocytes, admixed with CD68-positive histiocytes. All immunohistochemical profile of the tumor cells is summarized in Table 1.

**Table 1:** Immunohistochemical profile of the carcinoma components in our case of PC of the breast

**Positive**	**Negative**	
AE1/AE3	ER	D2-40
Cam5.2	PgR	CD31
CK5/6	HER2	CD34
EMA	E-cadherin	LCA
34βE12	β-catenin	bcl-2
vimentin	α-SMA	CD3
CD10	S-100 protein	CD8
CD4	p63	CD20
EGFR	calponin	CD79a
MIB-1 labeling index 30%	myogenin	c-KIT
	desmin	p53

Based on all these features, we indicated that these tumor cells were characteristic of epithelial differentiation, and finally made a diagnosis of PC of the breast associated with cyst formation. Final pathological stage was determined as pT2N0M0, stage IIA, according to the tumour node metastasis (TNM) system of the Union for International Cancer Control (UICC) 7th Edition [9].

## Discussion

The prognosis of the current PC patient might unusually appear better than that reported by Silver and Tavassoli [1], since it is likely that the final diagnosis was made at a relatively earlier stage of the tumors; even though follow-up period was not too long in this limited case report. Actually, our case has shown neither local recurrence nor metastases for 10 months after the operation. In contrast, Nguyen *et al.* recently have described that, although not all of these PCs behave badly, the presence of spindle cell sarcomatoid (metaplastic) components or the tumor size larger than 5 cm significantly leads to confer a significantly poor prognosis even in the early stage for PC of the breast [10]. In addition, as shown in this PC case, no immunohistochemical expression of E-cadherin and β-catenin, involved in cell adhesion and cell-cell interaction, very likely results in severe vessel permeation, advanced clinical stage, and worse outcome [11,12]. Hence, it would be critical for very early treatment against PC to establish an accurate preoperative diagnosis by fine needle aspiration cytology, the clinical utility of which in diagnosing breast tumors has been already generalized. The cytological characteristic features of PC of the breast seem to reflect the histopathological ones, exhibiting numerous individual bizarre and giant malignant cells with pleomorphic irregular nuclei, coarse chromatin, conspicuous nucleoli, very high mitotic rate, and relatively abundant cytoplasm in the background of possible inflammation and/or necrosis [6-8]. Indeed, when extensive necrosis is present without evidence of viable PC cells on cytology specimens, cytopathologists should exclude out the possibility of benign breast tumors, e.g., infarcted fibroadenoma [13]. On the other hand, we for the first time indicate that cohesive, three-dimensional and/or sheet-like clusters of highly malignant cells coexist on PC cytology. In fact, the cytological findings of this relatively new and extremely rare entity have never been well described or reviewed more recently. The cytodiagnostics might not need to come out with such a distinctive one, but should confirm malignancy in any case of PC of the breast. Despite that, a confident and accurate diagnosis of PC might be possible only on cytology specimens, owing to its cytomorphologically peculiar characteristics, adequate samplings, and/or accumulated experience. Furthermore, few previous papers proposed that, when the cytologic features of invasive ductal carcinoma with bizarre pleomorphic malignant giant cells were difficult to make an accurate and conclusive diagnosis, immunostaining for cytokeratins and EMA on cell blocks of aspirates would be very useful for the diagnosis of mammary PC [6,8]. Nevertheless, in cases with evidence of single bizarre giant cells and clusters of them, as shown here, cytopathologists should raise high possibility of PC as one of differential diagnoses, at the very least. Future studies are further needed.

It is possible that the present case report might be pathologically remarkable for three reasons at least: first, cystic cavity formation was uniquely recognized within the tissue of PC. Amongst the ‘true’ cases reported or retrospectively considered as PC of the breast in the English literatures [1,3,6-8,10,14], several PC tumors have displayed nodular or mass lesions with cystic change or cystic cavity formation [6,8,14], similar to the present case. We can hypothesize that the cystic cavity of mammary carcinoma would be formed via multiple processes: carcinoma cells initially develop in ductal wall and locally (i.e., intraductally) grow up in volume, and next block the ductal lumen possibly via their stromal involvements; and finally, the cystic cavity might gradually get larger with increased inner pressure and/or possible necrotic change. However, this hypothesis seems to be too speculative and not to be based on the histopathological features of our case. Nevertheless, since it remains to be elucidated whether PCs of the breast are prone to having cyst formation, it would be intriguing to study this topic.

Second, we have found the presence of ‘emperipolesis’ wherein surrounding inflammatory cells are engulfed by tumor giant cells of PC, very similar to pleomorphic giant cell carcinomas of the lung [15]. According to the recent, relatively large study of PCs of the breast by Nguyen *et al.*[10], many cases of them showed neoplastic ‘cannibalism’ of their tumor cells, but not of inflammatory cells. However, future thorough studies also will be further required to determine whether these features are genuinely characteristic in mammary PCs after histologically examining a larger number of PC cases.

Third, immunohistochemical analyses of not merely epithelial markers, including CK5/6 and 34βE12, but CD10 and vimentin were positively expressed in the tumor nests. Additionally, ER/PgR/HER2 were all triple-negative, possibly corresponding to basal-like type breast cancer [16]. Although there have been no large, detailed immunohistochemical studies of PC of the breast, to date, our data imply that those carcinoma cells might have potential squamous, myoepithelial, and/or basal-like phenotypes, and epithelial-mesenchymal transition (EMT), as well, supported by some published papers [14,16]. We might provide the possible evidence that PCs arise from ductal epithelial-myoepithelial cells, as a result of neoplastic transformation of outer supporting myoepithelial cells, as well as inner ductal epithelial cells [17,18]. However, since other myoepithelial markers examined, such as α-SMA, S-100 protein, p63, and calponin, were completely negative, this implication would be highly speculative and unlikely. First of all, pathological differential diagnosis of this mammary PC case could be metaplastic carcinoma, and indeed, metaplastic carcinoma of the breast include squamous cell carcinomas, spindle cell carcinomas, or carcinomas with mesenchymal differentiation [2]. In this context, not only by definition but from the above immunohistochemistry, it is very likely that PCs with metaplastic spindle and/or squamous differentiation are metaplastic carcinomas *per se*. Some confusion still exists in the newest WHO classification [2], and thus it could be very challenging that we pathologists strictly make a final diagnosis as PC of the breast.

It is possible that, based on these characteristic clinical and/or cytopathological features, as described above, PC of the breast might be a special new entity of the breast cancer, but not a rare variant of invasive carcinoma of no special type. In this context, the present case report could interest the scientific community, taken together with new cytological findings and specific recommendations for cytodiagnostics.

## Conclusion

We herein reported an extremely rare case of PC of the breast associated with cyst formation. The present case was tentatively made a diagnosis as high-grade invasive carcinoma, not otherwise specified, on the cytology specimens, even though its features seemed to be very characteristic. All cytopathologists should be aware that its cytomorphologically unique findings from extensively careful examination can induce one of differential diagnoses, and possibly a correct diagnosis.

## Consent

Written informed consent was obtained from the patient for publication of this case report and any accompanying images. A copy of the written consent is available for review by the Editor-in-Chief of this journal.

## Competing interests

The authors declare that they have no competing interests.

## Authors’ contributions

SY and AN participated in conception of the idea and writing of the manuscript. SY, AN, TT, HN, SK, SK, YN, KYW, SS and YS performed the cytopathological and immunohistochemical interpretation of the tumor tissue. All authors have read and approved the final manuscript.
